# Supplementary material for: Plasma Concentrations of Extracellular Vesicles Are Decreased in Patients with Post-Infarct Cardiac Remodelling
Source: Biology (Basel). 2021 Jan 30;10(2):97. doi: 10.3390/biology10020097 (PMC7910841; doi:10.3390/biology10020097)

Supplementary Materials

Plasma Concentrations of Extracellular Vesicles are Decreased in Patients with Post-Infarct Cardiac Remodelling

Aleksandra Gąsecka, Kinga Pluta, Katarzyna Solarska, Bartłomiej Rydz, Ceren Eyileten, Marek Postula, Edwin van der Pol, Rienk Nieuwland, Monika Budnik, Janusz Kochanowski, Miłosz J. Jaguszewski, Łukasz Szarpak, Tomasz Mazurek, Agnieszka Kapłon-Cieślicka, Grzegorz Opolski and Krzysztof J. Filipiak

Table S1. Eligibility criteria for the study.

Inclusion criteria	Exclusion criteria
• Age ≥ 18 years<br>• Informed consent to participate in the study<br>• First ST-elevation myocardial infarction (STEMI) or non-STEMI<br>• PCI with stent implantation<br>• Administration of the loading dose of clopidogrel (600 mg) prior to PCI	• Known coagulopathy<br>• Active pathological bleeding<br>• Known history of bleeding disorder<br>• Suspicion of intracranial haemorrhage<br>• Need for oral anticoagulation therapy<br>• Administration of GP IIb-IIIa antagonists<br>• Cardiogenic shock<br>• Severe chronic renal failure (eGFR < 30 mL/min)<br>• Severe liver insufficiency (Child-Pugh class C)<br>• Infectious disease<br>• Autoimmune disease<br>• Neoplasm<br>• Known pregnancy, breast-feeding, or intention to become pregnant during the study period

Abbreviations: AMI – acute myocardial infarction; CYP3A4 – cytochrome P450 izoenzyme 3A4; eGFR – estimated glomerular filtration rate; GP – glycoprotein; PCI – percutaneous coronary intervention

Table S2. Statistical estimates for prediction of left ventricle remodelling (LVR) by extracellular vesicles EVs from endothelial cells (CD146$^+$), erythrocytes (CD235a$^+$) and platelets (CD61$^+$).

EVs	AUC (95% CI)	p-value	Cut-off (per ml)	Sensitivity	Specificity	PPV	NPV
CD146	0.77 (0.60-0.95)	0.009	3.64 x 10^5	75%	70%	56%	84%
CD235a	0.75 (0.57-0.92)	0.018	1.67 x 10^7	83%	74%	63%	90%
CD61	0.76 (0.58-0.94)	0.014	1.68 x 10^8	83%	67%	59%	89%
**Combined**	**0.87 (0.73-1.00)**	**0.0004**	**As above combined**	**83%**	**87%**	**77%**	**91%**

Abbreviations: AUC: area under the curve, CI: confidence interval, PPV: positive predictive value, NPV: negative predictive value.

Table S3. Multivariate logistic regression model for prediction of left ventricle remodelling (LVR including extracellular vesicles (EVs) from endothelial cells ($CD146^+$), erythrocytes ($CD235a^+$) and platelets ($CD61^+$) along with clinical variables.

Variable	OR	95% CI		p-value
		Lower	Upper	
**Low $CD146^+$ EVs**	**8.243**	**1.253**	**54.219**	**0.028**
Age	1.011	0.920	1.111	0.819
Gender	0.265	0.028	2.497	0.246
AMI type	0.209	0.015	2.962	0.247
Peak troponin I	1.001	0.948	1.056	0.979
INR	19.662	0.001	1646212	0.607
**Low $CD235a^+$ EVs**	**17.854**	**2.300**	**138.561**	**0.006**
Age	0.989	0.902	1.085	0.989
Gender	0.265	0.028	2.497	0.328
AMI type	0.296	0.026	3.397	0.139
Peak troponin I	1.020	0.961	1.082	0.515
Creatinine	15.825	0.064	3914.155	0. 930
**Low $CD61^+$ EVs**	**21.457**	**0.805**	**572.067**	**0.067**
Age	0.971	0.853	1.105	0.657
Gender	0.239	0.013	4.511	0.339
AMI type	0.277	0.011	7.205	0.440
Peak troponin I	1.003	0.939	1.072	0.921
Platelet count	1.002	0.988	1.018	0.745
Hypertension	0.170	0.008	3.491	0.251

Abbreviations: AUC: area under the curve, CI: confidence interval

Figure S1. Concentrations of extracellular vesicles (EVs) measured with flow cytometry in platelet-depleted plasma prepared from patients with and without left ventricle remodeling (LVR) 6 months after AMI. A, B: EVs from activated platelets. C, D: EVs from leukocytes. E, F: EVs exposing phosphatidylserine (PS).

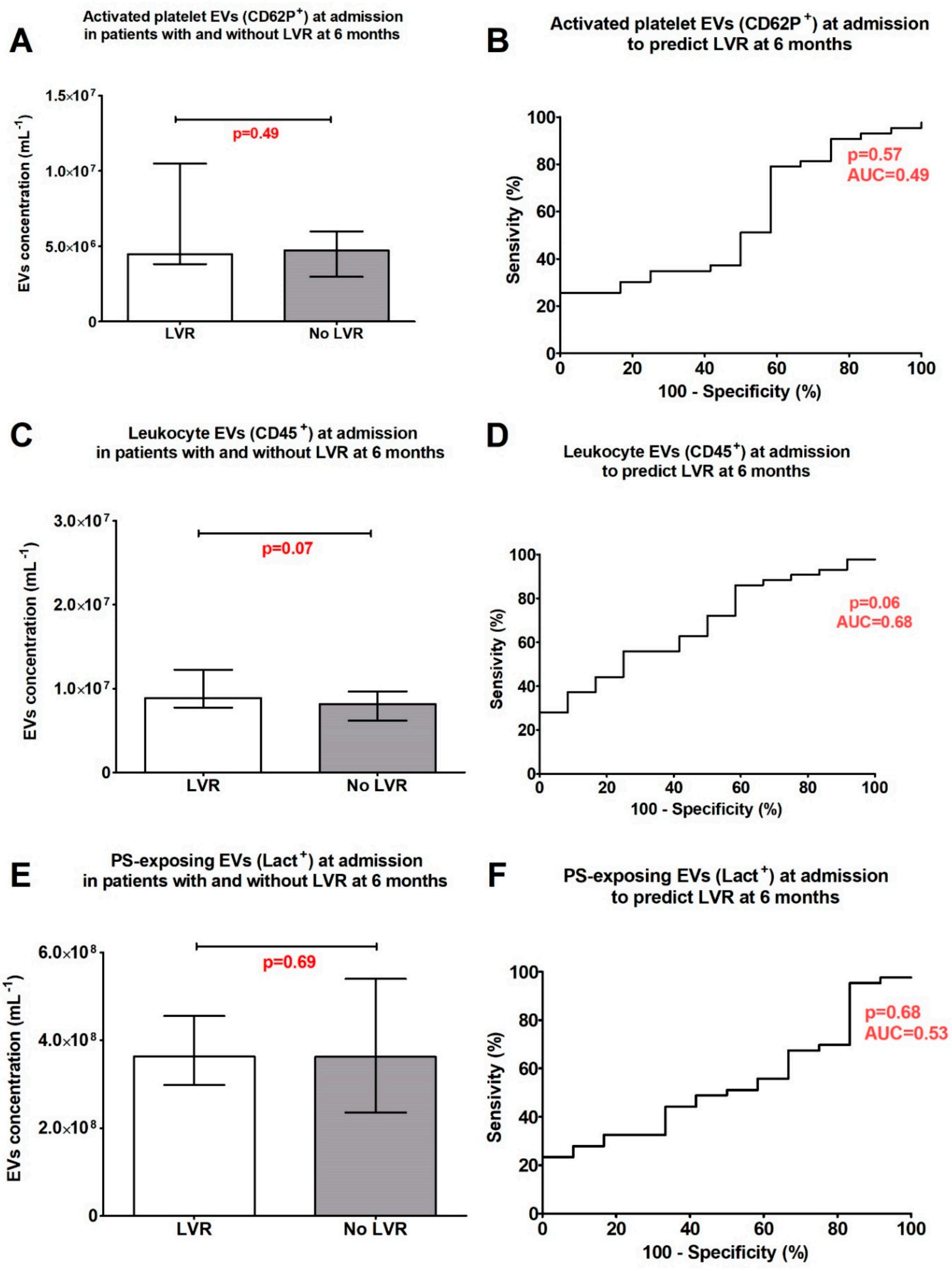

Figure S2. Concentrations of total particles and non-EV particles including chylomicrons (defined based on the differences in refractive index) measured with flow cytometry in platelet-depleted plasma prepared from patients with and without left ventricle remodelling (LVR) 6 months after AMI.

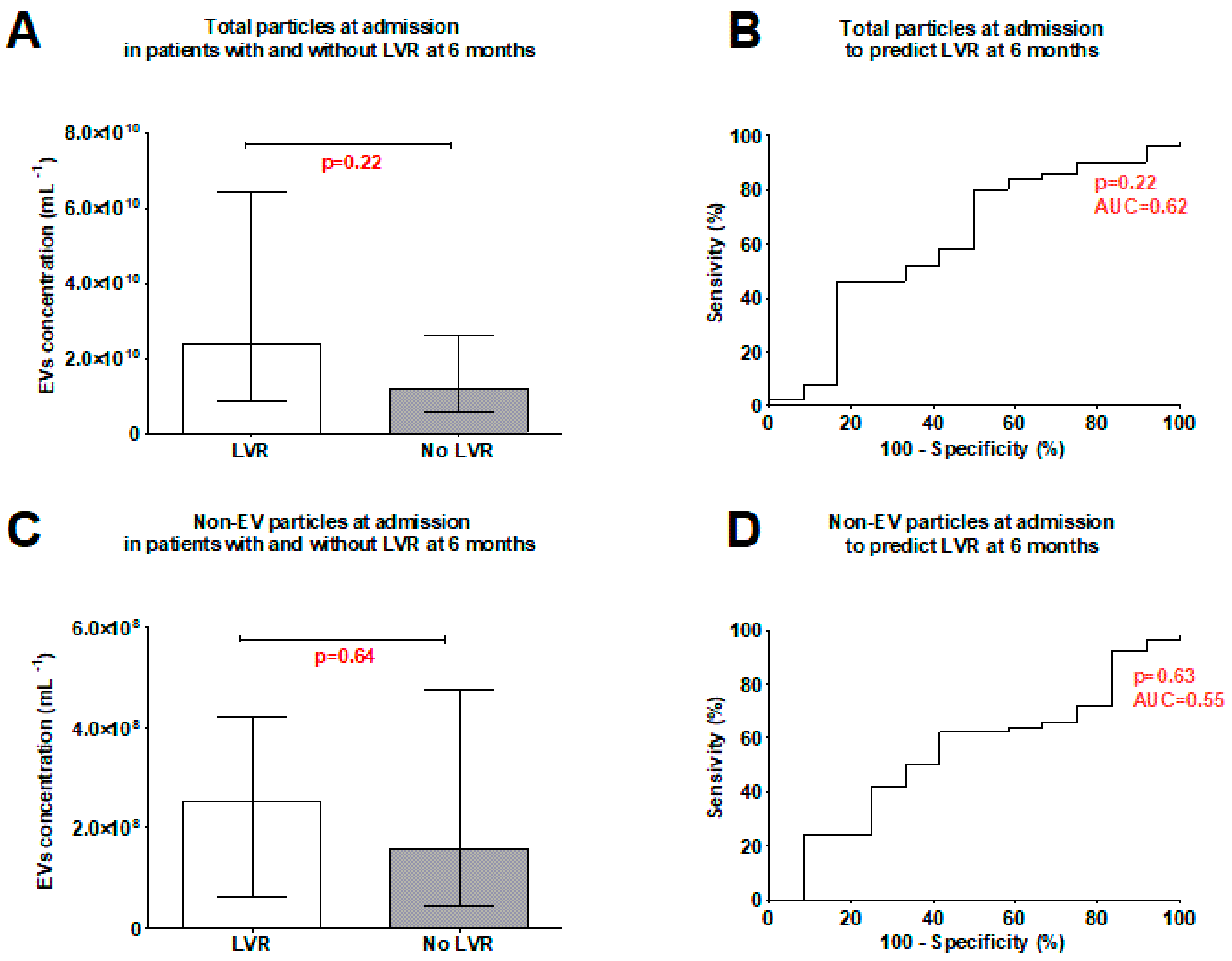

Supplement: Supplementary file 1 [file biology-10-00097-s001.pdf]
